# Detecting Microsatellite Instability in Endometrial, Colon, and Stomach Cancers Using Targeted NGS

**DOI:** 10.3390/cancers15205065

**Published:** 2023-10-20

**Authors:** Ulyana Boyarskikh, Andrey Kechin, Evgeniy Khrapov, Mikhail Fedyanin, Grigory Raskin, Marina Mukhina, Elena Kravtsova, Aleksey Tsukanov, Sergey Achkasov, Maksim Filipenko

**Affiliations:** 1 Institute of Chemical Biology and Fundamental Medicine, Siberian Division of the Russian Academy of Sciences, 630090 Novosibirsk, Russiakhrapovea@niboch.nsc.ru (E.K.); max@niboch.nsc.ru (M.F.); 2 State Budgetary Institution of Health Care of Moscow (Moscow Multidisciplinary Clinical Center “Kommunarka”), 142770 Moscow, Russia; 3 Dr. Berezin Medical Institute, 197758 St. Petersburg, Russia; rasking@list.ru (G.R.); mukhina@ldc.ru (M.M.);; 4 Ryzhikh National Medical Research Center of Coloproctology, 123423 Moscow, Russia

**Keywords:** microsatellite instability, endometrial cancers, targeted NGS

## Abstract

**Simple Summary:**

Among the genomic biomarkers, high microsatellite instability (MSI-H) has received FDA approval for selecting patients for immunotherapy with checkpoint inhibitors. In this regard, methods for accurate testing of MSI-H for a wide range of tumors are required. In this study, we developed an MSI testing method based on NGS of 81 microsatellite repeats and assessed its accuracy in 294 tumors from three cancer types. The method achieved the accuracy of classification of MSI-H and MSS tumors with AUC 0.99. The method can be integrated into the procedure of genomic profiling of tumors in standard clinic practice. Due to the use of a relatively large number of microsatellite markers, the method provides a quantitative assessment of MSI status and can be used in studies of the significance of MSI load as a prognostic marker of treatment outcome.

**Abstract:**

Purpose: To develop a method for testing the MSI based on targeted NGS. Methods: Based on the results of previous studies, 81 microsatellite loci with high variability in MSI-H tumors were selected, and a method for calculating the MSI score was developed. Using the MSI score, we defined the MSI status in endometral (162), colon (153), and stomach (190) cancers. Accuracy of the MSI scores was evaluated by comparison with MMR immunohistochemistry for 137 endometrium (63 dMMR and 74 pMMR), 76 colon (29 dMMR and 47 pMMR), and 81 stomach (8 dMMR and 73 pMMR) cancers. Results: Classification of MSS and MSI-H tumors was performed with AUC (0.99), sensitivity (92%), and specificity (98%) for all tumors without division into types. The accuracy of MSI testing in endometrial cancer was lower than for stomach and colon cancer (0.98, 87%, and 100%, respectively). The use of 27 loci only, the most informative for endometrial cancer, increased the overall accuracy (1.00, 99%, and 99%). Comparison of MSI score values in 505 tumors showed that MSI score is significantly higher in colon (*p* < 10^−5^) and stomach (*p* = 0.008) cancer compared with endometrial cancer. Conclusion: The MSI score accurately determines MSI status for endometrial, colon, and stomach cancers and can be used to quantify the degree of MSI.

## 1. Introduction

Deficiency in the mismatch repair (MMR) system is one of the genomic instability mechanisms in cancer. MMR-deficient tumors accumulate a large number of somatic mutations, including indels in microsatellite repeats, resulting in a specific molecular phenotype known as microsatellite instability (MSI) [1]. Indel mutations of microsatellites located in coding regions lead to the translation of unique peptides, a potential source of tumor-specific neoantigens. Because of the expression of neoantigens, tumors with MSI can be effectively recognized by the immune system. This explains the relatively favorable prognosis of tumors with MSI and their sensitivity to a recently approved type of immunotherapy (blockade of immune checkpoints with PD-1 inhibitors) [2,3]. Thus, high-grade MSI status (MSI-H) and MMR deficiency (dMMR) have recently been used in clinical practice as a marker of patient responsiveness to therapy.

The latest discovery fueled interest in the MSI phenomenon and contributed to the improvement of methods for its detection. The standard technique to determine MSI is based on PCR using different sets of marker microsatellites (such as Bethesda/NCI, Pentaplex, Idilla, etc.) [4,5]. The currently recommended PCR-MSI test includes a panel of five mononucleotide repeats (BAT-25, BAT-26, NR-21, NR-24, MONO-27) [6]. As an alternative, algorithms based on the NGS of a large number (from tens to several thousand) of mono- and dinucleotide microsatellite repeats are being developed.

Historically, PCR-MSI tests were developed primarily to determine MSI in the canonical cancers associated with Lynch syndrome, in particular colon cancer. Therefore, these tests tend to be more effective for detecting MSI events that occur in colon cancer. This is reflected in a higher false-negative rate of PCR-MSI testing of dMMR non-colorectal type tumors. Comprising IHC- and PCR-based methods for testing dMMR/MSI-H tumors showed a lower concordance for endometrial cancer than for colon cancer [7,8,9,10]. These findings formed the basis of ASCO’s strong recommendation to use MMR-IHC over MSI using PCR or NGS for patients with endometrial cancer being considered for PD1-inhibitor therapy [11]

Thus, there is a need for MSI testing methods across various types of tumors. Analysis of NGS data of the whole genome and the whole exome revealed the specificity of landscapes of variable microsatellite loci depending on the type of tumor [12,13,14]. The data obtained indicate the high importance of a combination of marker loci for the diagnostic performance of the MSI test.

NGS seems to be a strong analytical method for quantifying the variable alleles of microsatellite loci. Several groups have shown good results for pan-cancer testing of MSI using NGS of hundreds to thousands of microsatellite repeats that are accidentally sequenced along with target regions of large multigene panels [15,16]. However, such panels are not practical as a primary screening test due to the high cost, low sample throughput, and high DNA requirements.

Thus, there is a growing need for a small-scale NGS-based solution for accurate MSI detection with applicability across cancer types. Few targeted NGS panels for MSI assay have been described, but most have either been developed for colon cancer [17,18,19,20,21] or tested on small groups of different types of cancer [22,23,24].

In the present study, we developed a targeted NGS solution for accurate MSI detection and validated it in a cohort of endometrial, colon, and gastric cancer specimens with known MMR status.

## 2. Materials and Methods

### 2.1. Patients and Samples

This study was conducted in accordance with the Declaration of Helsinki and approved by the Institute of Chemical Biology and Fundamental Medicine (Protocol #8 from 7 July 2020). Written informed consent was obtained from patients for participating in this study. Samples were obtained from patients with colon (n = 153), endometrial (n = 162), or stomach (n = 190) cancer treated in N.N. Blokhin National Medical Research Center of Oncology and Dr. Berezin Medical Institute in 2020. Thirty-seven colorectal cancer samples and 17 endometrial cancer samples were obtained by endoscopy and curettage, respectively. The remaining samples were obtained after primary surgical tumor resection without prior neo-adjuvant therapy.

The formalin-fixed paraffin-embedded (FFPE) tumor blocks were sectioned and stained with hematoxylin and eosin (H&E). Tumor regions on H&E-stained slides were marked and the percentage of tumor cells was estimated. Samples containing at least 20% of tumor cells were selected for this study. DNA was extracted from marked regions separated by manual macrodissection from unstained 10 μm thick section. DNA isolation was carried out using the Cobas DNA Sample Preparation Kit (Roche, Basel, Switzerland).

Retrospective clinical IHC-MMR testing data were obtained for patients with colon (n = 76), endometrial (n = 137), and stomach (n = 81) cancer. More detailed data on samples are provided in Supplementary Table S1.

### 2.2. Immunohistochemistry for MMR Proteins

Analysis of expression of mismatch repair proteins was performed on 5 µm thick sections of the FFPE tumor blocks by using Ventana RTU (ready to use) antibody clones (MLH1, clone M1; MSH2, clone G2191129; MSH6, clone 44; PMS2, clone EPR3947) on a Ventana, BenchMark ULTRA IHC instrument (Ventana Medical Systems, Oro Valley, AZ, USA). CC1 (cell conditioning 1) pretreatment, and OptiView DAB IHC Detection Kit, according to Ventana protocol, were used for all antibodies. Tumors were classified as mismatch repair deficient (dMMR) if there was no nuclear staining of tumor cells for at least one of MLH1, MSH2, MSH6, and PMS2, in the presence of positive internal control. Presence of a nuclear immunolocalization was assessed by qualified pathologists using a light microscope, magnification ×200–400. Brown nuclear staining was considered as positive reaction. Degree of staining intensity was not evaluated. Complete absence of positive brown staining in tumor cells in the presence of brown staining in stromal and normal epithelial cells of adherent mucosa considered as negative reaction. Tumors with nuclear staining for all four markers were considered MMR proficient (pMMR). Representative photos of MMR proteins immunohistochemistry are shown in Supplementary Figure S1.

### 2.3. MSI Testing by Targeted NGS

MSI testing was performed by using the customized PCR-based NGS panel that included 81 microsatellite loci (Supplementary Table S2). To select microsatellite loci, we referred to the previously reported Ronald J. Hause et al. study, which, using tumor exomes from the Cancer Genome Atlas (TCGA), had defined a landscape of variable loci across 18 cancer types [8]. Based on the data from the study, we selected 108 mononucleotide repeats with significantly (qval < 10^6^) elevated frequency of instability in four types of MSI-H tumors (colon, rectum, endometrium, and stomach). Additionally, 14 microsatellites were selected from the database SelTarbase (http://seltarbase.org (accessed on 12 December 2020)) with the highest incidence of instability events (compared to that expected for this repeat length) in endometrial cancer. Several loci of these sets coincided, so the total number of candidate loci was 114. Primers for multiplex amplification of microsatellite sites were designed using the NGS-PrimerPlex tool [25]. The primer structures are available upon request. After the exclusion of loci with failed amplification or high variability in normal tissue (tested on 10 samples of leukocyte DNA), the final panel included 81 marker microsatellites.

To build the NGS library, DNA (at least 1 ng) was amplified in one multiplex PCR using Phusion High-Fidelity DNA Polymerase (New England Biolabs). The ratio of gene-specific primers was empirically selected to achieve better coverage uniformity. Amplification products were purified using a 1.5 volume of Agencourt AMPure XP magnetic beads (Beckman-Coulter). Labeling of samples with indices was carried out using 4 cycles of PCR with primers

i5-5′-aatgatacggcgaccaccgagatctacac(i5)acactctttccctacacgacgctcttccgatct-3′,

i7-5′-caagcagaagacggcatacgagat(i7)gtgactggagttcagacgtgtgctcttccgatct-3′

Containing unique combinations of TruSeq D501–508 and D701–712 indices i5 and i7 primers were annealed to the universal tails at the 5′-ends of the gene-specific primers and were extended to produce full-length, double-indexed molecules. The final PCR product after purification using a 0.8 volume of Agencourt AMPure XP was a library suitable for sequencing on any Illumina platform.

Normalized amplicon libraries were sequenced on a MiniSeq platform (Illumina) using a MiniSeq High Output Reagent Kit (300 cycles). Sequencing was performed to a target depth of at least 300 reads per amplicon (median, Q1, Q2: 650, 332, 1659). Indexing adaptor and PCR primer sequences were removed using Trimmomatic [26] and cutPrimers tools [27]. Reads were mapped to the human reference genome (hg19) using BWA (v. 0.7.12), and an inhouse Python script was used to count the number of reads with any indels in repeat sequences present within each of the microsatellite markers. After evaluating the proportion of reads with a different repeat length for each locus, the MSI score was calculated as the sum of these proportions. The Python script evaluating the percent of reads with insertions and deletions in the homopolymer tracts, and the example FASTQ-files with NGS reads for MSS and MSI samples can be accessed at https://github.com/aakechin/MSI-manuscript (accessed on 27 August 2023).

### 2.4. MSI Score Cutoff Determination

To establish a cutoff value for the classification of MSI-H and MSS tumors based on MSI score, we used fourfold cross-validation with MMR status (dMMR/pMMR) determined using IHC. Data from 294 samples with known MMR status were stratified and divided into 4 nearly same-size groups. Of the 4 groups, 1 was left to test the model, and the remaining 3 groups were used as a training set for MSI score cutoff detection. This procedure was repeated 4 times, and each of the groups was used once as a test group. As a result, the MSI score cutoff was selected from four MSI score thresholds calculated for each training set of samples. The procedures were performed by using the “train” function from the caret package in R (v. 4.2.3).

### 2.5. Statistical Analysis

The statistical analysis was performed with SciPy and sklearn Python modules; data visualization was carried out with seaborn and matplotlib Python modules. One-factor ANOVA test for independent measures including Tukey HSD was performed to determine the statistical significance of differences between the MSI scores for STAD, UCEC, and COAD cancers.

The confidence intervals for testing accuracy indicators were evaluated as for binomial proportion with “binomtest” and “proportion_ci” commands of the SciPy Python package. For the probability of success, the following MSI occurrences in the population were used for STAD, UCEC, and COAD: 10%, 30%, and 15%, respectively [28]. Calculating the distance between clusters in hierarchical cluster analysis was performed using the average linkage clustering method.

## 3. Results

### 3.1. Evaluation of NGS Panel Predictive Effectiveness

Two hundred and ninety-four tumor samples with known IHC-MRR status were used to evaluate the diagnostic performance of an 81-locus panel for NGS testing of MSI. These samples included cases of endometrial (63 dMMR and 74 pMMR cases), colon (29 dMMR and 47 pMMR cases), and stomach (8 dMMR and 73 pMMR cases) cancers. For each sample, we calculated the MSI score as a sum of proportions of differently sized alleles for each microsatellite repeat. Depending on the sample, MSI score values ranged from 4.9 to 31.1. To determine the threshold MSI score dividing tumors into MSI-H and MSS, we applied a fourfold cross-validation to the entire group of 294 samples. As a result, we found that with an MSI score threshold ranging from 9.5 to 10, tumors were classified using MSS and MSI-H with an accuracy of 96.8%. We chose 10 as the cutoff criterion for determining MSI status and used it to classify all 294 tumor samples.

The predictive performance of MSI score for MSI assay was evaluated using IHC detection of MMR proteins expression as a reference method (Table 1). MSI score-based classification of MSS and MSI-H cases regardless of cancer type was performed with an area under the ROC curve (AUC), sensitivity, and specificity of 0.99 (95% CI 0.97–1.00), 92% (95% CI 0. 85–96%), and 99% (95% CI 97–100%), respectively.

The specificity was close to 100% (98–100%) for all three types of cancer, in contrast to the sensitivity, which was 100% for colon and stomach cancers but was much lower for endometrial cancer, 87.3%.

### 3.2. Discordant Samples

Eight false-negative (FN) MSS cases of endometrial cancer and one false-positive (FP) MSI-H sample of colon cancer was found. We examined the discordant samples in more detail. For this purpose, we determined the level of *MLH1* promoter methylation and sequenced *MSH2*, *MSH6*, *MLH1*, and *PMS2* genes to determine somatic mutations (Table 2).

A false-positive MSI-H case of colon cancer is probably the result of a misinterpretation of the IHC test since somatic mutations in *MSH2* and *MSH6* were found in the DNA of the sample.

Of the eight FN cases, one case was characterized by a low level of *MLH1* promoter methylation and the absence of pathogenic variants in the MMR genes. These results reduce the credibility of the MMR test result. In one case, we were unable to perform additional testing. For the remaining cases, IHC-MMR status was confirmed by the findings of molecular DNA testing. In five cases, we found hypermethylation of the *MLH1* promoter and in one case, a somatic mutation in the *MLH1* gene. In the latter case, the proportion of NGS reads with mutations was 22%, while the proportion of tumor cells was 70%. This indicates the subclonal nature of the *MLH1* mutation, but IHC analysis did not reveal heterogeneity in the expression of *MLH1* protein. Such sample heterogeneity may be the cause of discordant test results.

Interestingly, we did not observe subclonal (secondary) mutations in the MMR genes for most IHC-dMMR cases. Perhaps this reflects the low frequency of MSI events in these tumors since such mutations (e.g., c.3261dupC in *MSH6*, our data) are often located in microsatellite repeats of the MMR genes and are a consequence rather than a cause of MMR function loss [29].

In addition, we assessed the effect of the content of tumor cells in the sample on the value of MSI score as a probable cause of low sensitivity. To determine the dependence of the MSI score on the tumor purity, we measured the MSI score in mixed samples containing 100, 80, 70, 50, 30, 20, and 0 percent of “tumor” DNA. To prepare these samples, we mixed DNAs isolated from tumor blocks and DNAs isolated from patient-matching blocks of normal tissue in appropriate ratios. Five sample pairs were examined, three for colon cancer (with MSI score values of 17.3, 20.2, and 26.1) and two for endometrial cancer (with MSI score values of 11.4 and 16.9). All five original samples contained about 80% of tumor cells.

**Figure 1:**
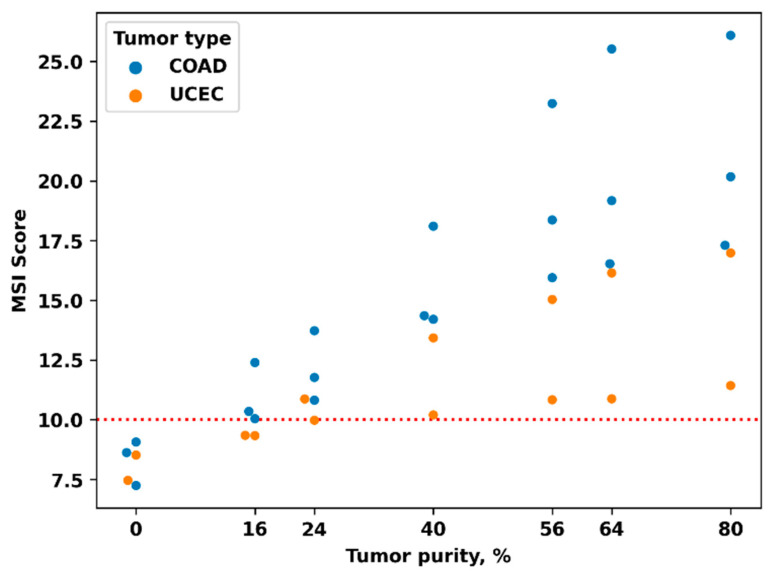
Dependence of MSI score on tumor purity. The plot shows that tumors with a low MSI degree pass the threshold MSI score (10) at a higher proportion of tumor cells. For UCEC, the content of tumor cells should be >30%.

We observed a borderline value of MSI score (9.98) for a sample with an initially low MSI score (11.4) and a tumor purity of about 25% (Figure 1). Therefore, a percentage of tumor cells ≥30% is required to achieve high accuracy of MSI testing based on MSI score. This is especially crucial for cancer types with presumably low MSI levels, particularly for endometrial cancer (see below for an explanation).

There are two samples where borderline tumor cell content (25 and 30%) could be the cause of the discordant test.

### 3.3. Reestimation of Marker Loci of the NGS Panel

**Figure 2:**
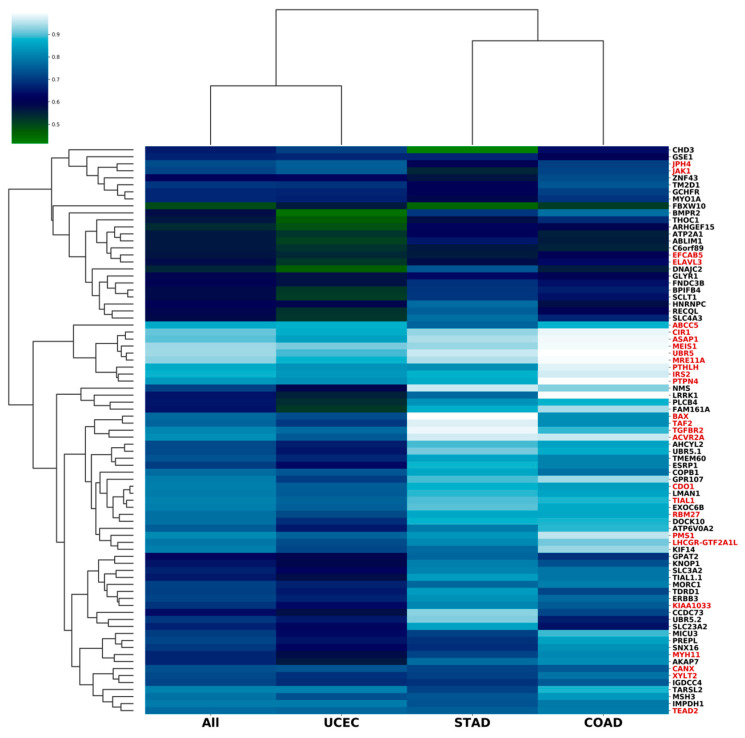
Heatmap showing AUC (area under the ROC curve) value for each of 81 microsatellite loci across stomach (STAD), endometrial (UCEC), and colorectal (COAD) cancer. Loci are organized by hierarchical clustering into groups with similar AUC patterns. AUCs were calculated for samples with a known IGH-MMR status. All loci are informative for predicting MSI status with AUC > 0.5. AUC values are systematically lower in UCEC compared to COAD and STAD. Twenty-seven loci included in the set of the “best” loci with higher predictive performance are highlighted in red.

We assumed that not all microsatellites of the NGS panel have a sufficiently high predictive performance. Therefore, we determined the AUC for individual loci, for all cases with known status of IHC MMR and clustered the loci according to the AUC value in different types of tumors (Figure 2, Supplementary Table S3). Our findings showed that for endometrial cancer, most of the loci had a lower AUC. The exception was *ABCC5*, *IMPDH1* (AUC was 0.87 and 0.79, respectively), and *GSE1*, *JAK1*, *JPH4,* and *CHD3* (AUC < 0.75).

Additionally, for each locus, we compared the variability in MSS and MSI cases separately for COAD, STAD, and UCEC tumors (Supplementary Table S4). Thus, we identified loci more frequently mutated in MSI tumors, specific to a particular type of cancer. The most specific loci were in the exons of genes *JAK1* and *CHD3* (for endometrial cancer); *BMPR2*, *ELAV3*, *GLYR1*, and *ZNF43* (for colon cancer); and *XYLT2* (for stomach cancer).

**Figure 3:**
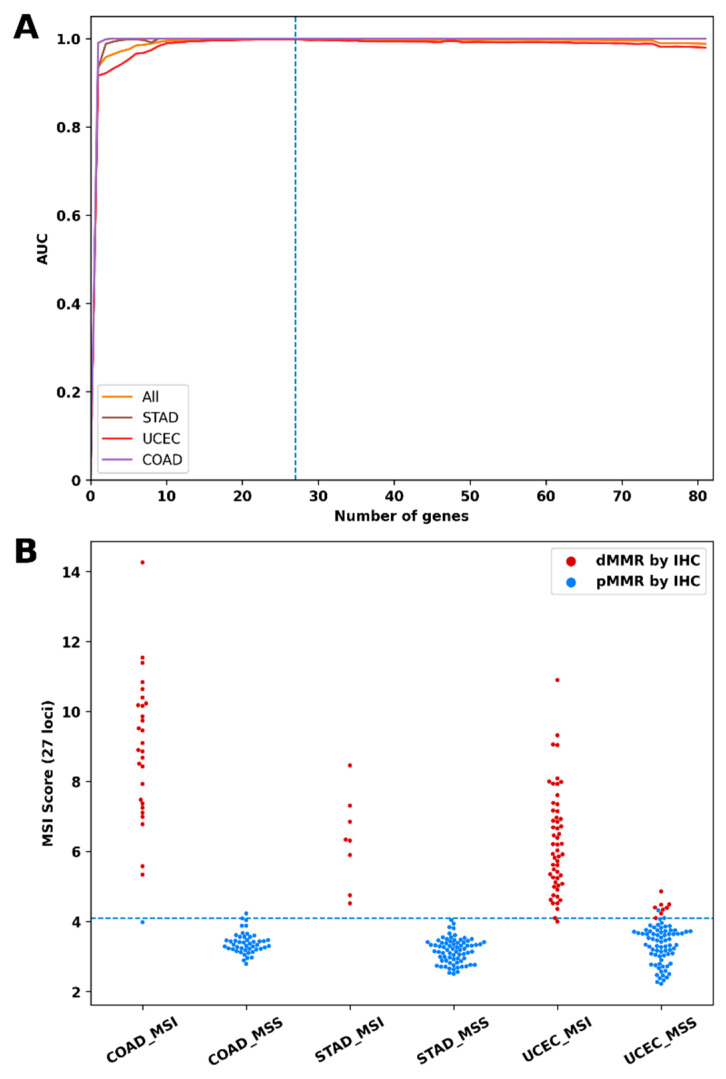
Development of an optimal set of microsatellite loci for accurate classification of MSS and MSI-H tumors. (**A**) The colored curves show the value of the AUC, which is achieved with an increasing number of microsatellite loci. AUC increases with the number of loci but plateaus with 27 loci. A further increase in the number of loci leads to a slight decrease in AUC. Sets with increasing numbers of loci were generated as follows: the loci were arranged in descending order of AUC for UCEC. The first set contained the first locus; the second locus was added to it and the AUC was recalculated. If the AUC increased, the locus was included, if not, the locus was excluded and the next one was added, etc. (**B**) Analysis using 27-MSI score (*Y*-axis) classified cases as MSS (<4) or MSI-H (≥4). *X*-axis denotes MSS/MSI-H classification based on 81-MSI score. Cases include 294 samples with known status of MSI: stomach (STAD, n = 81), endometrial (UCEC, n = 137), and colorectal (COAD, n = 76) cancers. Red points indicate dMMR samples and blue points indicate pMMR samples. 27-MSI score calculated based short set of 27 “best” loci; 81-MSI Score calculated based on original set of 81 loci. It can be seen that most of the FN (according to the 81-MSI Score) samples of the UCEC have the 27-MSI score, >4, and should be classified as dMMR.

Since some of the loci had a high AUC value (close to the AUC of the whole NGS panel) and, in addition, these loci were grouped into clusters with similar patterns of variability (Supplementary Figures S2–S4), we concluded that the 81-loci NGS panel could be redundant. To determine the optimal number of loci, we plotted the dependence of the predictive performance on the number of loci tested (Figure 3A). For this task, the loci were arranged in descending order of AUC for endometrial cancer. Then, we recalculated the AUC for sets containing an increasing number of loci: loci were added to the set one by one in the specified order; if the AUC increased, the locus was included in the set, otherwise, the locus was skipped and the next one was added. The entire set of 294 samples with known IHC-MMR status was used to calculate AUC. With this approach, the most informative loci for endometrial cancer were included in the set first.

Figure 3A shows that the sets containing more than 27 loci had no additional advantages; moreover, the predictive performance of sets with 70 or more loci was slightly lower. The 27 loci that were included in the set are indicated in Figure 1. Interestingly, several tumor-specific loci were included in this set despite the relatively low values of the AUC, for example, *ELAV3* (0,58), *XYLT2* (0.72), and *JAK1* (0.71).

Finally, we recalculated the MSI scores using data on the frequency of variable alleles for these 27 loci only and, by cross-validation with the IHC-based MMR, specified the cutoff criterion as 4. As a result, using the updated MSI Score, the tumors were classified using MSS and MSI-H with higher accuracy (Table 3, Figure 3B).

### 3.4. Degree of MSI in Different Cancer Types

Previously, whole-exome sequence data indicating that the number of MSI events is highly variable within and across tumor types were obtained [13]. We calculated the MSI score as the frequencies of all alleles with indels in marker mononucleotide repeats; hence, the value of the MSI score correlated with the intensity of the mutation process in repeats (in other words, the degree of MSI).

**Figure 4:**
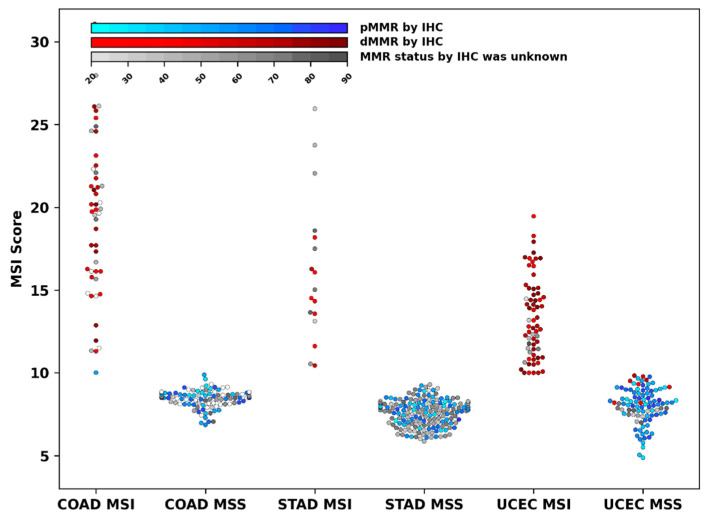
Swarm plot showing the distribution of MSI score for stomach (STAD, n = 190), endometrial (UCEC, n = 162), and colorectal (COAD, n = 153) cancer. Red points indicate samples with deficient IHC-MMR status (dMMR). Blue points indicate samples with proficient IHC-MMR status (pMMR). Grey points indicate samples not tested using IHC. The color gradient reflects the proportion of tumor cells. Transparent points indicate samples with unknown tumor cell content. MSS and MSI tumors were classified using MSI score with cutoff criteria—10. MSI score was calculated based on NGS data of 81 loci.

To assess the level of MSI depending on the type of tumor, we determined the MSI score for 211 additional samples using an NGS panel of 81 loci. The MSI score values for 505 samples of the combined set are shown in Figure 4. We compared the MSI score of all samples that were classified as MSI-H (MSI Score ≥ 10) for COAD, STAD, and UCEC tumors.

Our analysis revealed high variability in MSI score between tumor types, with MSI score values distributed continuously over a wide range (Figure 4). Pairwise comparison of MSI scores of tumors with MSI-H (MSI score > 10) showed significant differences in their mean values between the COAD and UCEC tumors (19.5, 95% CI [18.1, 20.9] vs. 13.3, 95% CI [12.75, 13.9] with *p* < 10^−5^) and between the STAD and UCEC tumors (16.2, 95% CI [14.1, 18.3] vs. 13.3, 95% CI [12.75, 13.9] with *p* = 0.008). It is important that the sample sets compared were, on average, with a similar content of tumor cells. There was also no significant correlation between the percentage of tumor cells in individual samples and the corresponding MSI score.

## 4. Discussion

Since the Food and Drug Administration approved anti-PD-1 immunotherapy for the treatment of unresectable or metastatic, MSI-high/dMMR tumors in 2017, irrespective of tumor type, the number of patients tested for MSI/MMR has been increasing constantly. Although the MSI-PCR test is the gold standard for MSI assays in the clinical setting, there is a growing interest in NGS panel-based MSI testing.

In our research, we focused on the development of an NGS test suitable for testing MSI in different types of cancer, especially in endometrial cancer. Endometrial cancer is characterized by the highest incidence of MSI; however, the diagnosis of MSI for it is less accurate than, for example, for colon cancer [11].

To date, few variants of MSI tests based on small targeted NGS panels validated for endometrial cancer have been described [22,23,24]. Yosuke Hirotsu et al. [22] used an amplicon-based NGS panel of 76 loci to determine MSI across 25 types of cancer (181 cases, of which 7 were endometrial cancers) with specificity (91.3%) and sensitivity (100%). The Jason Willis group [23] developed a 99-loci hybridization-based NGS panel for MSI detection using cell-free DNA sequencing. Clinical validation of the method was performed for 25 types of cancer (949 cases, mainly the colon, lung, and stomach cancers, of which about 10 cases were endometrial cancers). The authors used MSI-PCR and NGS as the main reference methods. For 112 cases with known IHC-MMR status, specificity, and sensitivity were 94% and 52%, respectively. However, the task of testing cell-free plasma DNA is much more difficult than DNA from FFPE blocks. Another interesting solution using single-molecule molecular inversion probe capture to enrich the library with target sequences was proposed by Adam Waalkes et al. [24]. Using a panel of 111 loci, the authors defined MSI across colorectal (n = 68, 100% sensitivity and specificity), prostate (n = 33, 100% sensitivity and specificity), and endometrial cancers (n = 43, 95.8% diagnostic sensitivity and 100% specific). The use of smMIPs and UMIDs provided the analytical sensitivity of the test (at least 1% MSI-positive cells). However, this approach has not yet found broad application in routine diagnostics.

Here, we developed a small NGS panel of microsatellite loci and an algorithm for determining MSI status and evaluated the predictive performance of the NGS panel for MSI assay for pMMR and dMMR tumors of COAD (76), STAD (81), and UCEC (137). The panel included loci known from previous reports to be affected by MSI in various types of cancer. We used an algorithm to calculate the MSI score as the pooled mutation frequency of microsatellite repeats targeted. This workflow was developed for tumor samples and does not need patient-matched normal samples or any other baseline normal controls.

In terms of the above method, the NGS test described by Yosuke Hirotsu et al. [22] is the closest to our solution and differs only in the set of markers and the MSI score evaluation algorithm (MSIcall) using a baseline control of the normal profile of microsatellite repeats. However, our study provides a more reliable estimate of the accuracy of MSI testing in endometrial cancer.

We examined two sets of microsatellite repeats: the original panel, including 81 loci, where the threshold value of the MSI score was 10, and an updated panel, including 27 loci, the threshold value of MSI Score was 4. Both assays showed better predictive performance in COAD and STAD tumors than in UCEC tumors. All loci in the original panel were informative for MSS and MSI-H classification, with AUCs greater than 0.5. Despite this, the exclusion of loci with relatively low AUC increased the predictive characteristics of the MSI score for endometrial cancer (AUC value increased from 0.98, CI 95% [0.94–1.00] to 1.00, CI 95% [0.97–1.00]). In contrast, both the whole panel and its short versions (with 5–27 of top loci) with approximately equal efficiency predicted the MSI status of gastroenterological cancers. We received evidence that the panel including 81 loci is largely redundant. However, we cannot conclude the optimal loci set, since the 27 loci left in the panel short version were selected using the same initial data as it was trained with; hence, the data could be overfitted. Therefore, 27-locus panel validation on an independent set of clinical samples is required.

Since the AUC values of all individual loci were systematically lower in UCEC, we supposed that any common (non-tumor-specific) model for determining MSI status would be less effective for UCEC than COAD and STAD, regardless of the combination of loci.

Our findings are consistent with other studies. In a recent report, the MSI status of 40 endometrial and 138 colon adenocarcinomas sequenced with MSK-IMPACT was detected with a sensitivity of 93% and 100%, respectively [15]. The mSINGS assay of the 15-locus NGS panel achieved 75% for UCEC and 94% for COAD tumors [30].

Latham et al. observed that up to 29% of Lynch syndrome-associated and dMMR endometrial cancers did not have MSI-H (by the MSK-IMPACT MSIsensor assay); instead, they had intermediate MSI sensor score (MSI-I) values. With that, 96% of all the Lynch syndrome-associated colon cancers were MSI-H and only 4% were MSI-I [31].

The relatively low efficiency of DNA assays for classifying MSI-H endometrial cancer is probably due to the lower frequency of indels in microsatellite repeats. This fact is confirmed by recent studies. I. Cortes-Ciriano et al. found diversity in the number of exonic MSI events: median MSI events per exome was 290 for colorectal cancer and 126 for endometrial cancer; in addition, 17% of MSI-H endometrial tumors had <50 MSI events/exome [13].

In this study, we used the MSI score that presents the sum frequency of all MSI events in eighty-one microsatellites and hence characterizes the degree of MSI to some extent. Consistent with the above observations, our findings showed a significant difference in median MSI scores between UCEC and COAD/STAD tumors. Moreover, of the sixty-two cases of dMMR UCEC tumors, fourteen had subthreshold MSI score values (from 8 to 10).

The significance of MSI degree as a quantitative prognostic marker for immunotherapy with checkpoint inhibitors remains to be elucidated. Recent studies in a mouse model showed that a low degree of MSI was insufficient for cell sensitivity to anti-PD-1 therapy despite the loss of MMR function. Only advanced high-degree MSI resulted in the manifestation of an immunogenic phenotype responsive to anti-PD-1 treatment [32].

Ronald J. Hause et al. also observed a correlation between survival outcomes and the overall burden of unstable microsatellites and suggested that MSI may be a continuous rather than binary phenotype [12].

These data raise the question of whether the degree of MSI score is a quantitative prognostic metric for response to therapy and patient outcome. Thus, methods for diagnosing not only the MSI status, but also its degree with a numerical criterion, will become in demand in research and, soon, possibly in clinical practice.

## 5. Conclusions

We showed that MSI status can be accurately determined from NGS data of 81 microsatellite markers using the MSI score across COAD, STAD, and UCEC tumors. The accuracy of the NGS panel of 27 loci may be higher, but additional validation on an independent set of samples is required to confirm this. Moreover, the MSI score provides an MSI degree of quantification, which may be useful for research on the MSI degree as a prognostic marker.

## Figures and Tables

**Table 1:** Performance of 81-MSI score for classification MSS and MSI-H tumors versus IHC as reference test in stomach (STAD), endometrial (UCEC), and colorectal (COAD) cancer. 81-MSI score was calculated based on NGS of 81 loci, the threshold value of MSI score was 10.

Samples	IGH	MSI-H ≥10	MSS <10	AUC (95% CI)	Sensitivity (95% CI)	Specificity (95% CI)
All n = 294	dMMR pMMR	92 1	8 193	0.99 (0.97–1.00)	92% (85–96%)	99% (97–100%)
STAD n = 81	dMMR pMMR	8 0	0 73	1.00 (0.96–1.00)	100% (63–100%)	100% (95–100%)
UCEC n = 137	dMMR pMMR	55 0	8 74	0.98 (0.94–1.00)	87% (76–94%)	100% (95–100%)
COAD n = 76	dMMR pMMR	29 1	0 46	1.00 (0.95–1.00)	100% (88–100%)	98% (89–100%)

**Table 2:** Detailed description of discordant samples.

Tumor Type	Content of Tumor Cells (%) *	NGS-MSI	IHC-MMR	*MLH1* Promotor Methylation	NGS-MMR (Gene, Mutation Position, and Mutation Rate)
UCEC	25 **	MSS	MLH1-; PMS2-	neg	wt
UCEC	50	MSS	MLH1-; PMS2-	pos	PMS2 (c.1351dupA, 0.09)
UCEC	40	MSS	MLH1-; PMS2-	ND ***	ND
UCEC	30	MSS	MLH1-	pos	wt
UCEC	50	MSS	MLH1-; PMS2-	pos	wt
UCEC	65	MSS	MLH1-; PMS2-	pos	wt
UCEC	55	MSS	MLH1-; PMS2-	pos	wt
UCEC	70	MSS	MLH1-; PMS2-	neg	MLH1 (c.884 + 1G > A, 0.22)
COAD	50	MSI	pMMR	neg	MSH2 (c.1588G > T, 0.48)

* The percentage of cells was only approximate; in some cases, such as metastases or samples after curettage, this assessment was difficult. ** The sample was a metastasis to a lymph node from the uterus; *** ND—no data, additional analysis could not be performed due to insufficient sample.

**Table 3:** Performance of 27-MSI score for classification MSS and MSI-H tumors versus IHC as reference test in stomach (STAD), endometrial (UCEC), and colorectal (COAD) cancer. 27-MSI score was calculated based on of NGS of the “best” 27 loci; the threshold value of MSI Score was 4.

Samples	N	AUC (95% CI)	Sensitivity (95% CI)	Specificity (95% CI)
All	294	1.00 (0.99–1.00)	99% (95–100%)	99% (96–100%)
STAD	81	1.00 (0.96–1.00)	100% (63–100%)	100% (95–100%)
UCEC	137	1.00 (0.97–1.00)	98% (91–100%)	99% (93–100%)
COAD	76	1.00 (0.95–1.00)	100% (88–100%)	98% (89–100%)

## Data Availability

The data presented in this study are available in this article and supplementary material.

## Supplementary Materials

The following supporting information can be downloaded at: https://www.mdpi.com/article/10.3390/cancers15205065/s1. Supplementary Figure S1: Representative photos of MMR proteins immunohistochemistry. Supplementary Figure S2–S4: Clustering of microsatellite loci depending on variability. Table S1: The samples description. Table S2: List of loci. Table S3: AUC for individual loci. Table S4: Loci variability between MSS and MSI tumors.
