# Supplementary material for: Detecting Microsatellite Instability in Endometrial, Colon, and Stomach Cancers Using Targeted NGS
Source: Cancers (Basel). 2023 Oct 20;15(20):5065. doi: 10.3390/cancers15205065 (PMC10605658; doi:10.3390/cancers15205065)
Supplement: Supplementary file 1 [file cancers-15-05065-s001.zip › Supplementary Figures S1-S4.pdf]

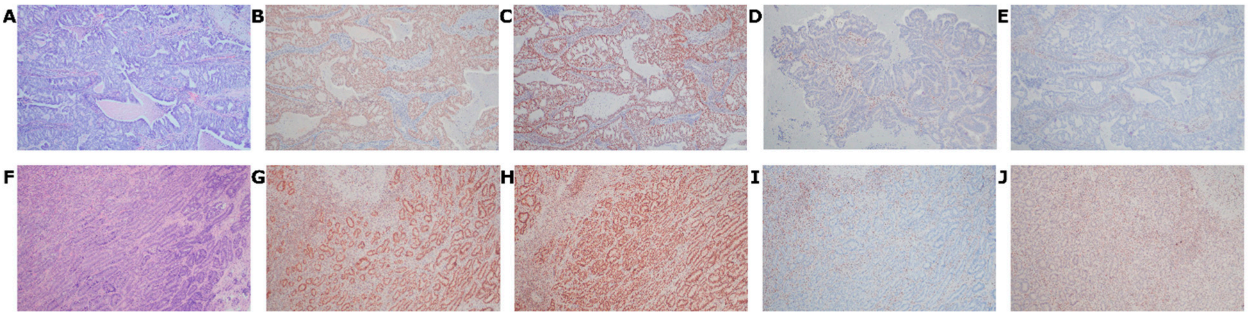


**Figure S1**: A - Endometrioid carcinoma. x100; B - Positive MSH2 reaction in tumor cells. x100; C - Positive MSH6 reaction in tumor cells. x100; D - Negative PMS2 reaction in tumor cells. x100; E - Negative MLH1 reaction in tumor cells. x100; F - Intestinal adenocarcinoma. x 100; G - Positive MLH1 reaction in tumor cells. x 100. H - Positive PMS2 reaction in tumor cells. x 100. I - Negative MSH6 reaction in tumor cells. x100. J - Negative MSH2 reaction in tumor cells. x100. A, F - haematoxylin and eosin stain; B-E, G-J – immunohistochemical stains.

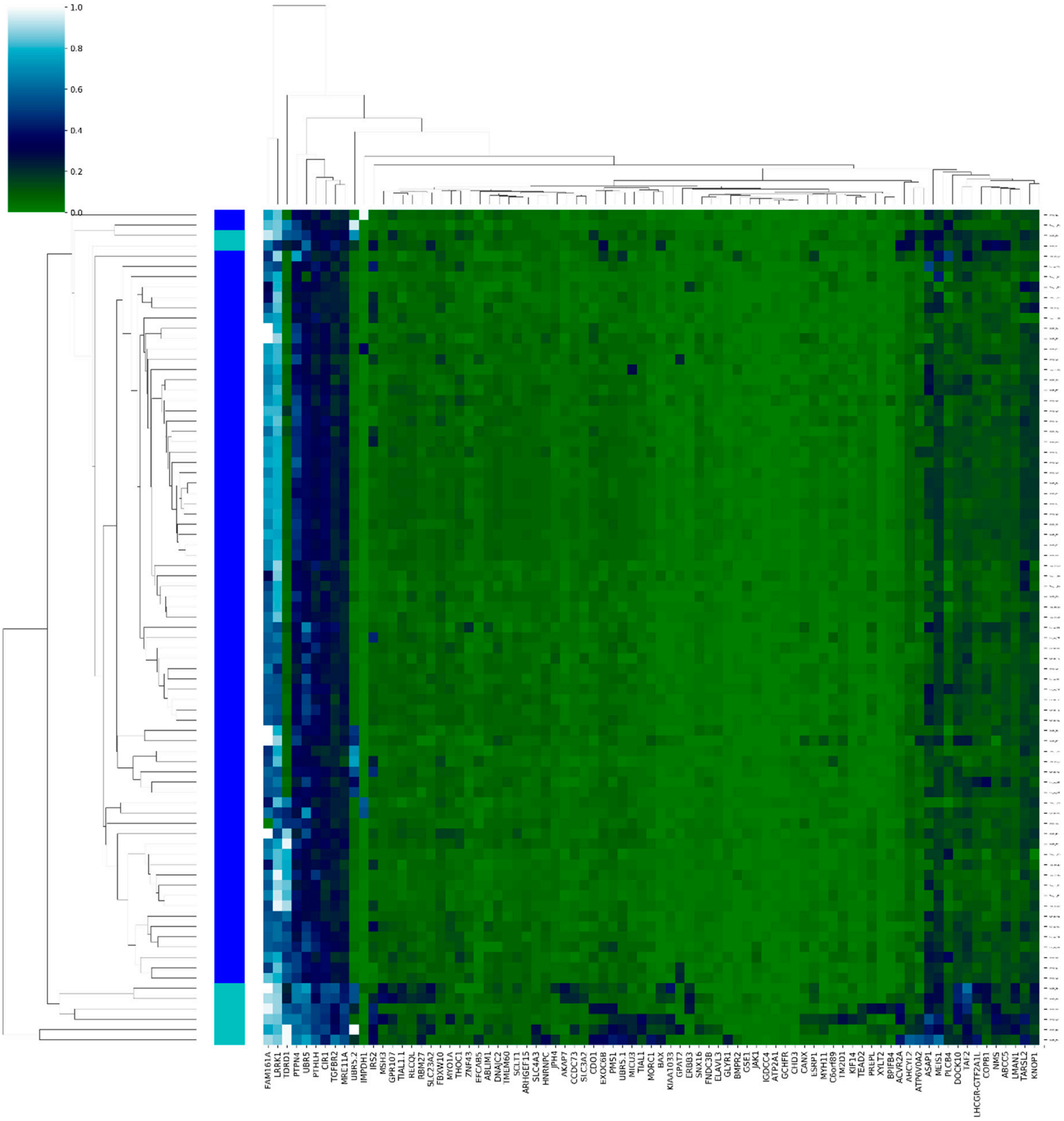

**Figure S2**: Heatmap indicating the proportions of loci with high variability in the stomach cancers with proficient MMR (pMMR) and deficient MMR (dMMR) status. Loci are organized by hierarchical clustering into groups with similar variability patterns.

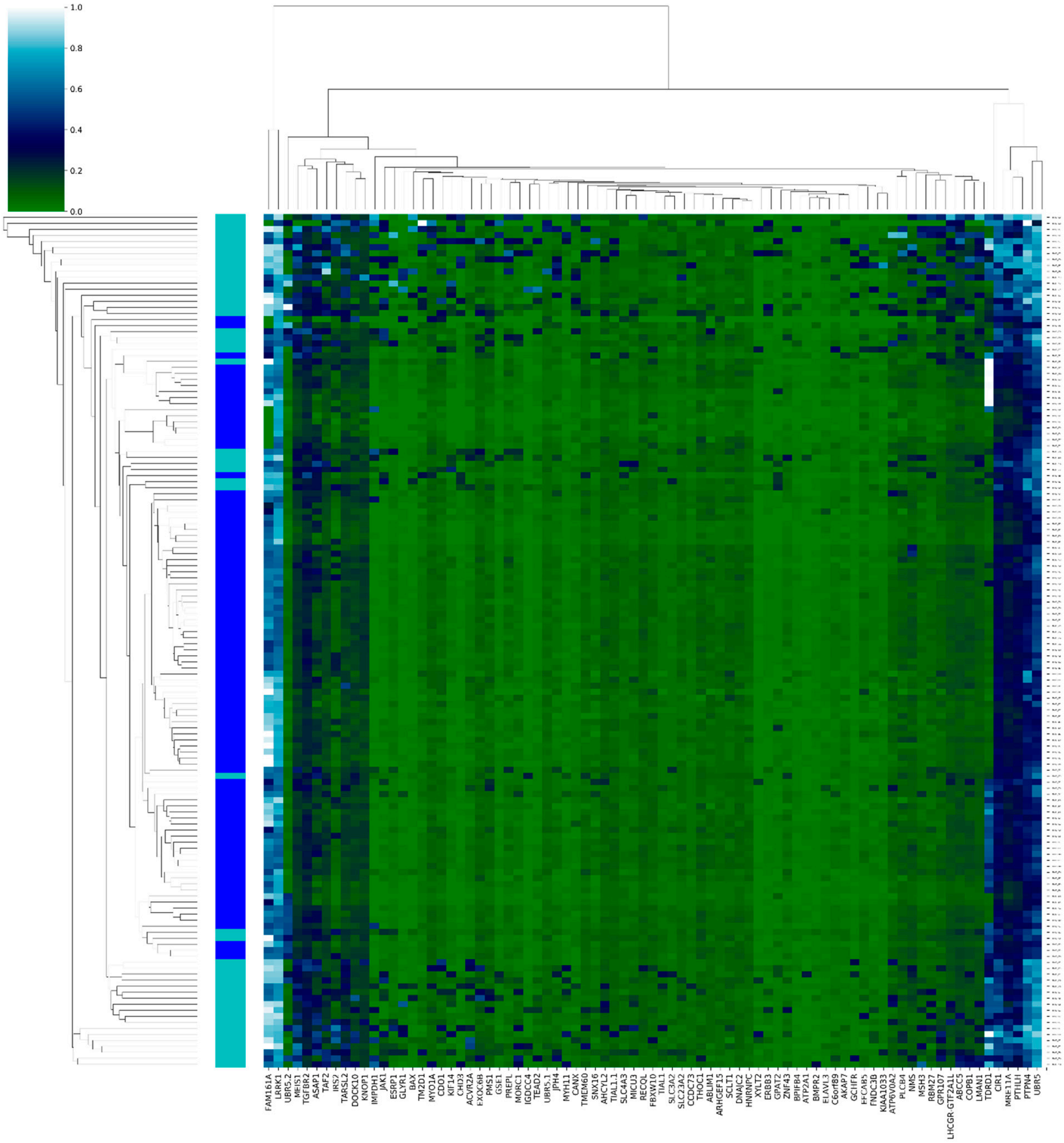


**Figure S3**: Heatmap indicating the proportions of loci with high variability in the endometrial cancers with proficient MMR (pMMR) and deficient MMR (dMMR) status. Loci are organized by hierarchical clustering into groups with similar variability patterns.

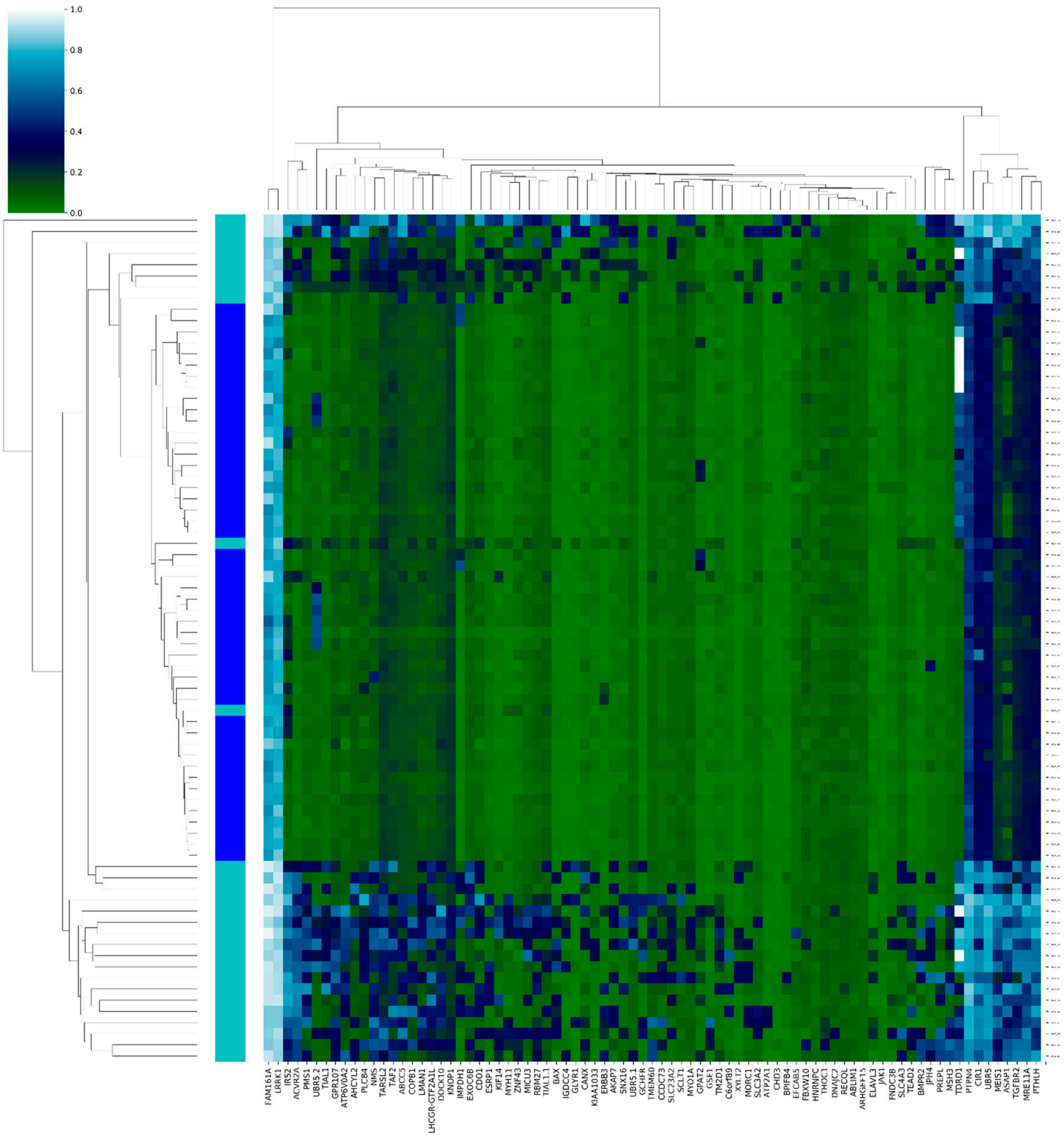


**Figure S4**: Heatmap indicating the proportions of loci with high variability in the colon cancers with proficient MMR (pMMR) and deficient MMR (dMMR) status. Loci are organized by hierarchical clustering into groups with similar variability patterns.
